# Hippocampal Subfield Volumetry: Differential Pattern of Atrophy in Different Forms of Genetic Frontotemporal Dementia

**DOI:** 10.3233/JAD-180195

**Published:** 2018-06-19

**Authors:** Martina Bocchetta, Juan Eugenio Iglesias, Marzia A. Scelsi, David M. Cash, M. Jorge Cardoso, Marc Modat, Andre Altmann, Sebastien Ourselin, Jason D. Warren, Jonathan D. Rohrer

**Affiliations:** a Dementia Research Centre, Department of Neurodegenerative Disease, Institute of Neurology, University College London, London, UK; b Translational Imaging Group, Centre for Medical Image Computing, University College London, London, UK

**Keywords:** Genetic frontotemporal dementia, hippocampal subfields, magnetic resonance imaging, volumetry

## Abstract

**Background:**

Frontotemporal dementia (FTD) is a heterogeneous neurodegenerative disorder, with a strong genetic component. Previous research has shown that medial temporal lobe atrophy is a common feature of FTD. However, no study has so far investigated the differential vulnerability of the hippocampal subfields in FTD.

**Objectives:**

We aimed to investigate hippocampal subfield volumes in genetic FTD.

**Methods:**

We in6/2/2018vestigated hippocampal subfield volumes in a cohort of 75 patients with genetic FTD (age: mean (standard deviation) 59.3 (7.7) years; disease duration: 5.1 (3.4) years; 29 with *MAPT*, 28 with *C9orf72*, and 18 with *GRN* mutations) compared with 97 age-matched controls (age: 62.1 (11.1) years). We performed a segmentation of their volumetric T1-weighted MRI scans to extract hippocampal subfields volumes. Left and right volumes were summed and corrected for total intracranial volumes.

**Results:**

All three groups had smaller hippocampi than controls. The *MAPT* group had the most atrophic hippocampi, with the subfields showing the largest difference from controls being CA1-4 (24–27%, *p* < 0.0005). For *C9orf72*, the CA4, CA1, and dentate gyrus regions (8–11%, *p* < 0.0005), and for *GRN* the presubiculum and subiculum (10–14%, *p* < 0.0005) showed the largest differences from controls.

**Conclusions:**

The hippocampus was affected in all mutation types but a different pattern of subfield involvement was found in the three genetic groups, consistent with differential cortical-subcortical network vulnerability.

## INTRODUCTION

Frontotemporal dementia (FTD) is a clinically, pathologically, and genetically heterogeneous neurodegenerative disorder. Around a third of patients with FTD have an autosomal dominant mutation in one of three genes: microtubule-associated protein tau (*MAPT*), progranulin (*GRN*), and chromosome 9 open reading frame 72 (*C9orf72*) [1]. Although traditionally described as characteristic of Alzheimer’s disease, medial temporal lobe atrophy is commonly seen in FTD [2] with the hippocampus often strikingly affected, particularly in carriers of mutations in the *MAPT* gene [3, 4], where volume loss occurs 15 years before expected onset [5], and there is a faster rate of atrophy compared with other genetic forms of FTD [6, 7].

The hippocampus is composed of different cytoarchitectonic subfields, which have specialized functions and distinctive connections [8, 9]. Recently, advanced parcellation methods based on atlases built from ultra-high resolution scans of histology sections have led to the development of post-processing techniques of high-resolution magnetic resonance (MR) scans that allow visualization and measurement of the hippocampal subfields *in vivo* [10]. Given the recent availability of this method, the differential vulnerability of the hippocampal subfields across the genetic forms of FTD has so far not been investigated. This study aimed to look into this further with the hypothesis that the three genetic groups would have different patterns of subfield involvement.

## METHODS

We reviewed the UCL Dementia Research Centre FTD database to identify all patients who were symptomatic carriers of a mutation in the *MAPT*, *GRN,* or *C9orf72* genes and who had also undergone a volumetric T1-weighted MR scan. 75 patients were identified: 29 *MAPT* (28 with behavioral variant FTD, bvFTD [11], and one with progressive nonfluent aphasia, PNFA [12]), 28 *C9orf72* (24 bvFTD, 2 PNFA, 2 FTD with associated motor neuron disease, FTD-MND), and 18 *GRN* (11 bvFTD, 5 PNFA, and 2 primary progressive aphasia not otherwise specified, PPA-NOS [13]). 97 cognitively normal subjects, with a similar age to the patients and with a usable volumetric T1-weighted MRI, were identified as controls. The study was approved by the local ethics committee and written informed consent was obtained from all participants. The study was conducted in accordance with the Helsinki Declaration of 1975.

MRIs were acquired from 1993 to 2017 with scanners from three different manufacturers: 69 on 1.5T Signa MRI scanner (GE Medical systems, Milwaukee, WI, TR = 12 ms, TI = 650 ms, TE = 5 ms, acquisition matrix = 256 × 256, spatial resolution = 1.5 mm), 85 on 3T Trio MRI scanner (Siemens, Erlangen, Germany, TR = 2200 ms, TI = 900 ms, TE = 2.9 ms, acquisition matrix = 256 × 256, spatial resolution = 1.1 mm), and 18 on 3T Prisma MRI scanner (Siemens, Erlangen, Germany, TR = 2000 ms, TI = 850 ms, TE = 2.93 ms, acquisition matrix = 256 × 256, spatial resolution = 1.1 mm). We reviewed the MRIs to make sure we excluded individuals with moderate to severe vascular disease or space occupying lesions.

T1-weighted volumetric MRI scans were first bias field corrected and whole-brain parcellated using the geodesic information flow (GIF) algorithm [14], which is based on atlas propagation and label fusion. Volumes of the whole hippocampus and of 12 hippocampal subfields were subsequently segmented using a customised version of the module available in FreeSurfer 6.0 [10], to adapt the output of GIF to the FreeSurfer format. We focused on the following subregions: hippocampal tail, cornu ammonis 1 (CA1), CA2/3, CA4, subiculum, presubiculum, and the granule cell layer of the dentate gyrus (DG). We decided to exclude from the analysis the hippocampus– amygdala transition area, the parasubiculum, the molecular layer of the hippocampus, the fimbria and the hippocampal fissure, as they were too small, not reliably delineated on T1-weighted images, or white matter tissue.

Left and right volumes were summed and corrected for total intracranial volumes (TIV). Volumes are expressed as a percentage of TIV, computed with SPM12 v6470 (Statistical Parametric Mapping, Wellcome Trust Centre for Neuroimaging, London, UK) running under Matlab R2014b (Math Works, Natick, MA, USA) [15]. All segmentations were visually checked for quality by an expert in hippocampal segmentation and none was excluded. We also investigated asymmetry by calculating an Asymmetry Index (AI), defined as the absolute difference between the left and right total hippocampal volumes in relation to the total bilateral volume: |(Left – Right)|/(Left + Right). The volumetric differences between groups were computed as follow: (Mean of Controls – Mean of Genetic Group)/Mean of Controls*100.

Statistical analyses were performed on subfield volumes (as percentage of TIV) and AI in SPSS software (SPSS Inc., Chicago, IL, USA) v22.0, between control and patient groups, using the ANCOVA test adjusting for scanner type, gender and age. When comparing each volume and AI between different patient subgroups (in pairs), we also adjusted for disease duration. For the subfield analysis, results were corrected for multiple comparisons (Bonferroni’s correction), and we considered them significant at *p* < 0.007.

## RESULTS

Demographic and clinical data are reported in Table 1. The mean disease duration for the whole FTD group at the time of the scan was 5.1 years (standard deviation 3.4) with an average age at onset at 54.2 (7.7). There was no significant difference in age between FTD and controls (*p* = 0.052, *t*-test), or for scanner type (*p* = 0.297, Chi square test), but there were more males in the FTD group than in the control group (61% versus 44%, *p* = 0.027, Chi square test). Across the different genetic FTD groups, there was no difference for scanner type nor gender (*p* = 0.281 and 0.322, Chi square test). There was also no difference across the different genetic FTD groups and controls (*p* = 0.247 and *p* = 0.070, Chi square test). However, there was a significant difference in disease duration (*p* = 0.010, ANOVA), with *C9orf72* having the longest and *GRN* the shortest duration, and in age (*p* = 0.001, ANOVA), with *MAPT* being the youngest group.

**Table 1:** Demographic and clinical variables for the FTD patients and controls. Values denote mean (standard deviation) or n (%)

Groups	*n*	Gender, male	Age at scan (y)	Disease Duration (y)	Clinical Diagnosis
controls	97	43 (44%)	62.1 (11.1)	–	—
*MAPT*	29	17 (59%)	55.3 (7.9)	5.5 (3.3)	28 bvFTD, 1 PNFA
*GRN*	18	9 (50%)	62.2 (6.4)	3.0 (2.6)	11 bvFTD, 5 PNFA, 2 PPA-NOS
*C9orf72*	28	20 (71%)	61.5 (6.7)	6.0 (3.6)	24 bvFTD, 2 PNFA, 2 FTD-MND

**Fig. 1:**
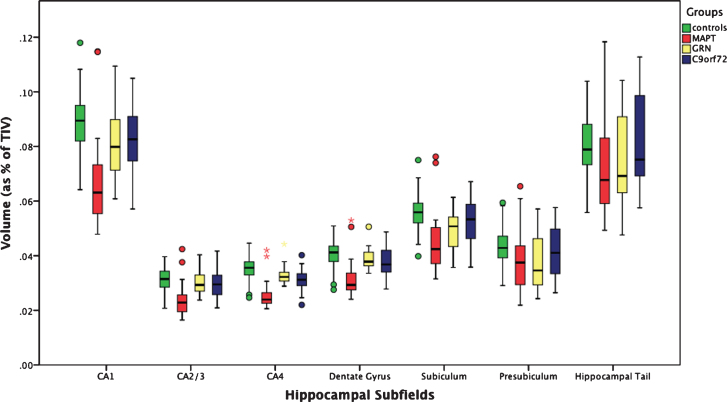
Volume of the hippocampal subfields as a percentage of total intracranial volume in 97 controls and 75 patients with genetic FTD (29 *MAPT*, 18 *GRN*, and 28 *C9orf72*).

**Fig. 2:**
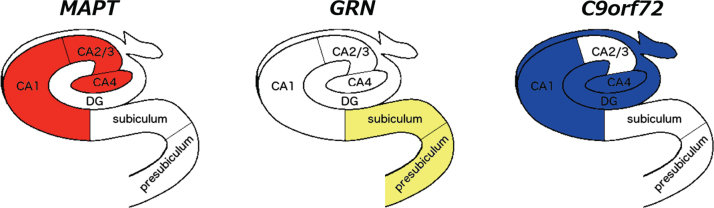
Differential volumetric patterns for the three main genetic FTD forms when compared to controls. The most affected subfields for each gene are shown in color on a coronal representation of the hippocampus at the level of the body. CA, cornu ammonis; DG, granule cell layer of the dentate gyrus.

The whole hippocampus was significantly smaller in all three genetic groups when compared to controls (*p* < 0.0005, ANCOVA), with the *MAPT* group showing the highest difference in volume (19%; *GRN*: 8%; *C9orf72*: 5%) (Table 2). For all the subfields, *MAPT* showed a strong and highly significant difference from controls. In *MAPT* carriers, the most affected subfields were the CA regions (27–24%, *p* < 0.0005), followed by the dentate gyrus (23%, *p* < 0.0005), while the hippocampal tail was the least affected (9%, *p* < 0.0005). The subiculum and presubiculum were the most affected subfields in *GRN* carriers (10 and 14%, *p* < 0.0005), while for *C9orf72* CA4 (11%), the dentate gyrus and CA1 (both 8%, *p* < 0.0005) were the most affected (Figs. 1 and 2 and Table 2).

**Table 2:** Volumetry of hippocampal subfields in 97 healthy non-carrier controls and 75 genetic FTD patients

	Controls (97)		*MAPT* (29)		*GRN* (18)		*C9orf72* (28)		*MAPT* versus Controls		*GRN* versus Controls		*C9orf72* versus Controls	
Structure	Mean	SD	Mean	SD	Mean	SD	Mean	SD	*p*-value	difference	*p*-value	difference	*p*-value	difference
Whole hippocampus	0.480	0.047	0.391	0.090	0.443	0.068	0.455	0.073	**<0.0005**	**19%**	**<0.0005**	**8%**	**<0.0005**	**5%**
CA1	0.089	0.010	0.067	0.017	0.081	0.012	0.082	0.012	**<0.0005**	**24%**	**<0.0005**	**9%**	**<0.0005**	**8%**
CA2/CA3	0.032	0.004	0.024	0.006	0.031	0.004	0.030	0.005	**<0.0005**	**24%**	0.054	3%	0.042	6%
CA4	0.036	0.004	0.026	0.005	0.034	0.004	0.032	0.004	**<0.0005**	**27%**	**<0.0005**	**6%**	**<0.0005**	**11%**
Dentate gyrus	0.041	0.005	0.032	0.007	0.040	0.004	0.038	0.005	**<0.0005**	**23%**	**<0.0005**	**4%**	**<0.0005**	**8%**
Subiculum	0.056	0.006	0.045	0.011	0.050	0.008	0.053	0.008	**<0.0005**	**19%**	**<0.0005**	**10%**	**0.001**	**6%**
Presubiculum	0.044	0.006	0.038	0.010	0.038	0.010	0.042	0.010	**<0.0005**	**13%**	**<0.0005**	**14%**	**0.001**	**5%**
Hippocampal tail	0.080	0.010	0.073	0.017	0.076	0.018	0.082	0.017	**<0.0005**	**9%**	**<0.0005**	**5%**	**0.003**	–**3%**

Values denote mean and standard deviation (SD) volumes as % of total intracranial volume (TIV) or difference (%). *p*-values denote significance on ANCOVA test. Bold represents a significant difference between groups after correcting for multiple comparisons.

When directly comparing the three genetic subgroups, the *MAPT* group showed significantly lower volumes in the whole hippocampus than *GRN* (13%, *p* < 0.0005, ANCOVA) and *C9orf72* (16%, *p* = 0.008) (Table 3). For the subfields, the *MAPT* group showed significantly smaller CA1, CA2/3, CA4, dentate gyrus (20–29% difference) and subiculum (11%) than *GRN,* and significantly smaller CA1, CA2/3, CA4 (22–23%) and dentate gyrus (18%) than *C9orf72*. No differences were found between the *C9orf72* and *GRN* groups. Supplementary Table 1 shows additional volumetric results for the hippocampal subfields separated for the left and right hemisphere showing a similar pattern.

**Table 3:** Comparisons of volumetry of the hippocampal subfields in 75 genetic FTD patients

	*MAPT* versus *GRN*		*MAPT* versus *C9orf72*		*GRN* versus *C9orf72*	
Structure	*p*-value	difference	*p*-value	difference	*p*-value	difference
Whole hippocampus	**0.001**	–**13%**	**0.008**	–16%	0.764	–3%
CA1	**<0.0005**	–**20%**	**<0.0005**	–**22%**	0.968	–1%
CA2/CA3	**<0.0005**	–**27%**	**<0.0005**	–**23%**	0.445	3%
CA4	**<0.0005**	–**29%**	**<0.0005**	–**22%**	0.131	6%
Dentate gyrus	**<0.0005**	–**24%**	**0.001**	–**18%**	0.394	5%
Subiculum	**0.005**	–**11%**	0.013	–16%	0.669	–4%
Presubiculum	0.146	1%	0.309	–10%	0.193	–11%
Hippocampal tail	0.089	–5%	0.144	–13%	0.807	–8%

*p*-values denote significance on ANCOVA test. Bold represents a significant difference between groups after correcting for multiple comparisons.

**Fig. 3:**
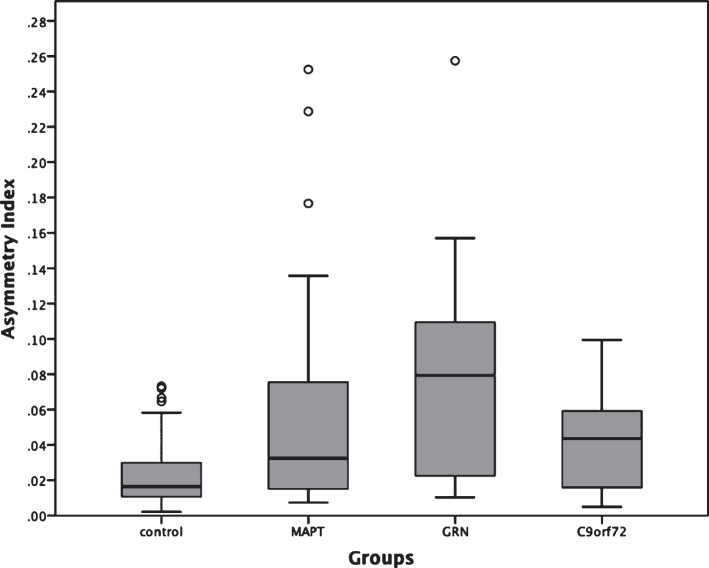
Asymmetry Index for the whole hippocampus in 75 genetic FTD patients (29 *MAPT*, 18 *GRN* and 28 *C9orf72*) and 97 controls. *MAPT*, *C9orf72*, and *GRN* versus controls: *p* < 0.0005; *MAPT* versus *GRN*: *p* = 0.064; *MAPT* versus *C9orf72*: *p* = 0.146; *GRN* versus *C9orf72*: *p* = 0.027 (ANCOVA).

All three genetic groups showed significantly more asymmetry of the whole hippocampus than controls (mean: 0.020, standard deviation: 0.017; *p* < 0.0005, ANCOVA). The *MAPT* group (0.058, 0.067) did not show any significant difference when compared to *GRN* (0.079, 0.061) and *C9orf72* (0.039, 0.029) (*p* = 0.064 and *p* = 0.146, respectively), while the *GRN* group showed significantly more asymmetry than *C9orf72* (*p* = 0.027, ANCOVA) (Fig. 3).

We performed a comparison of hippocampal subfields volumes in the different scanners in the control group adjusting for age and found the following results reported on Supplementary Table 2: overall volumes tend to be slightly smaller on 1.5T scanner than on 3T ones with the range of difference from 3–9%.

## DISCUSSION

We used an advanced automated segmentation method based on atlases built from ultra-high resolution scans of histological sections to extract volumes of hippocampal subfields in a large cohort of patients with genetic FTD. Those with *MAPT* mutations were the most affected group overall, a finding in line with the literature [3, 5, 7]. However, we also showed a pattern of differential involvement: the *MAPT* group showed an impairment in the hippocampus proper (formed by the CA subfields), *C9orf72* in the dentate gyrus and CA1/4, and *GRN* in the subiculum and presubiculum.

Anatomical and imaging studies of hippocampal subfield connectivity to other cortical and subcortical regions provide insight into the differential involvement of the FTD genetic disorders [8, 9, 16]. The *MAPT *group showed greater involvement of the anterior and central regions of the hippocampus compared with the hippocampal tail. These regions are connected to the amygdala, nucleus accumbens, cingulate, and the medial prefrontal and orbitofrontal cortex [9, 16–18], a network linked to the regulation of emotions and goal-directed behaviour as part of the limbic system, previously described to be affected in *MAPT* mutations [6]. The *GRN* group showed the greatest involvement of the subiculum and presubiculum. A recent intrinsic connectivity study of the hippocampal subfields [19] showed that the subiculum connects to the lateral and medial parietal lobes and striatum as well as frontal regions, which have been described as key atrophic areas in *GRN* mutations [5, 20]. In the same study, CA4 and dentate gyrus (most affected in *C9orf72)* were connected with temporal and posterior cortical areas [19], similar to the early regions of involvement seen in this mutation group [5, 20]. This hypothesis of differential network involvement and our results are in line with pathological studies: tau deposition is extensively found in the hippocampus and other limbic structures in the early phases of FTD due to *MAPT* mutations [21]; dipeptide repeat proteins (DPRs), together or without TDP-43 deposition, are found in the CA subregions in *C9orf72*, and DPRs are also found in the cerebellum and the thalamus; while TDP-43 accumulates in the hippocampus and the cortex in *GRN*.

The *GRN* carriers were the most asymmetric group, consistent with previous literature highlighting the striking asymmetry in many cases with such mutations [6]. However, we also found that the *MAPT* and *C9orf72* groups were significantly more asymmetric than controls albeit to a lesser extent than the *GRN* group. While the majority of studies of *MAPT* and *C9orf72* have shown no difference in symmetry at the level of individual hemispheres, there is commonly subtle asymmetry in individual lobes or subcortical structures which is lost at a hemispheric level–the extent of such differences or their biological basis has yet to be studied in depth. As previously reported in the literature [23], the asymmetry index in the control group is non-zero with the right hippocampus being bigger than the left. Larger studies will be required to understand the asymmetrical involvement of the individual subfields.

This study has a number of limitations. It includes different scanners (three manufacturers, two different magnetic fields: 1.5T and 3T) with slightly different MRI sequence types, and age and disease duration differences between the genetic groups. We took into account these variables and corrected for them in the statistical model, but this cannot completely remove some of the heterogeneity in this genetic dataset. Moreover, we used an automated method to extract the subfield volumes, which is not as accurate as their segmentation on brain tissue postmortem, nor as their manual segmentation on MR images. After reviewing the segmentations, we decided to exclude from the analysis the smallest subfields which were not reliably delineated on T1 MR imaging, particularly in this cohort who had atrophic hippocampi. However, nonetheless, the larger subfields are consistently and accurately defined using this methodology providing *in vivo* volumetry of hippocampal subfields, with the automated nature allowing analysis of large cohorts. Manual segmentation on these large datasets would be very time-consuming and labor-intensive, as it would require extensive anatomical knowledge and may take several hours per MR scan for even an expert manual rater.

The whole hippocampus is affected in all genetic forms of FTD [2, 5], as we have also shown here. The advantage of subfield delineation as we have done here (rather than focusing on the whole hippocampal volume) is the ability to better understand group differences and therefore distinguish between the different genetic forms and their intrinsic networks: limbic system in *MAPT*, temporal and posterior areas in *C9orf72* and fronto-parietal-striatum in *GRN*. Being able to investigate the hippocampal subfields with their clearly different projections will be helpful in providing further insights in disentangling the differences among the genetic forms of FTD.

Future studies, using functional and diffusion MR imaging, will be needed to investigate the different connections of these hippocampal subfields in each genetic form of FTD in more detail. Moreover, it will be important to investigate subfield volumetry both at the presymptomatic stage (through cohorts such as the Genetic FTD Initiative [5]) and longitudinally, to understand the differential involvement of the hippocampus over the course of the disease.

## Supplementary Material

Supplementary Tables
