# Supplementary material for: Hippocampal Subfield Volumetry: Differential Pattern of Atrophy in Different Forms of Genetic Frontotemporal Dementia
Source: J Alzheimers Dis. 2018 Jun 19;64(2):497–504. doi: 10.3233/JAD-180195 (PMC6027942; doi:10.3233/JAD-180195)
Supplement: Supplementary Tables [file jad-64-jad180195-s001.docx]

**Supplementary Table 1.** Volumetry of left and right hippocampal subfields in 97 healthy non-carrier controls and 75 genetic FTD patients (29 *MAPT*, 18 *GRN*, and 28 *C9orf72*). Values denote mean and standard deviation (SD) volumes as % of total intracranial volume (TIV) or difference (%). p-values denote significance on ANCOVA test. **Bold represents a significant difference between groups after correcting for multiple comparisons.**

|  | **Control (97)** | | ***MAPT* (29)** | | ***GRN* (18)** | | ***C9orf72* (28)** | | ***MAPT* versus Controls** | | ***GRN* versus Controls** | | ***C9orf72* versus Controls** | | ***MAPT* versus *GRN*** | | ***MAPT* versus *C9orf72*** | | ***GRN* versus *C9orf72*** | |
| --- | --- | --- | --- | --- | --- | --- | --- | --- | --- | --- | --- | --- | --- | --- | --- | --- | --- | --- | --- | --- |
| **Structure** | **Mean** | **SD** | **Mean** | **SD** | **Mean** | **SD** | **Mean** | **SD** | **p-value** | **%** | **p-value** | **%** | **p-value** | **%** | **p-value** | **%** | **p-value** | **%** | **p-value** | **%** |
| **Left** | | | | | | | | | | | | | | | | | | | | |
| **Whole hippocampus** | 0.238 | 0.024 | 0.188 | 0.051 | 0.215 | 0.044 | 0.223 | 0.037 | **<0.0005** | **21%** | **<0.0005** | **9%** | **<0.0005** | **6%** | 0.006 | -15% | 0.009 | -19% | 0.892 | -4% |
| **CA1** | 0.044 | 0.005 | 0.033 | 0.009 | 0.040 | 0.008 | 0.040 | 0.007 | **<0.0005** | **25%** | **0.003** | **9%** | **<0.0005** | **8%** | **<0.0005** | **-22%** | **0.001** | **-23%** | 0.992 | -1% |
| **CA2‎/CA3** | 0.015 | 0.002 | 0.012 | 0.003 | 0.015 | 0.003 | 0.015 | 0.003 | **<0.0005** | **23%** | 0.312 | 2% | 0.159 | 3% | **<0.0005** | **-26%** | **0.001** | **-25%** | 0.883 | 1% |
| **CA4** | 0.018 | 0.002 | 0.013 | 0.003 | 0.016 | 0.003 | 0.016 | 0.002 | **<0.0005** | **27%** | **<0.0005** | **8%** | **<0.0005** | **11%** | **<0.0005** | **-26%** | **<0.0005** | **-23%** | 0.928 | 3% |
| **Dentate gyrus** | 0.020 | 0.002 | 0.016 | 0.004 | 0.019 | 0.003 | 0.019 | 0.003 | **<0.0005** | **23%** | **<0.0005** | **6%** | **<0.0005** | **8%** | **0.001** | **-23%** | **0.001** | **-20%** | 0.956 | 2% |
| **Subiculum** | 0.028 | 0.003 | 0.022 | 0.006 | 0.025 | 0.005 | 0.026 | 0.004 | **<0.0005** | **22%** | **<0.0005** | **11%** | **0.001** | **7%** | 0.013 | -14% | 0.01 | -19% | 0.775 | -4% |
| **Presubiculum** | 0.022 | 0.004 | 0.018 | 0.006 | 0.018 | 0.005 | 0.020 | 0.005 | **<0.0005** | **20%** | **<0.0005** | **19%** | **<0.0005** | **9%** | 0.115 | -1% | 0.364 | -13% | 0.264 | -13% |
| **Hippocampal tail** | 0.040 | 0.005 | 0.034 | 0.010 | 0.037 | 0.010 | 0.040 | 0.008 | **<0.0005** | **14%** | **<0.0005** | **8%** | **0.002** | **1%** | 0.108 | -8% | 0.185 | -16% | 0.81 | -8% |
| **Right** | | | | | | | | | | | | | | | | | | | | |
| **Whole hippocampus** | 0.243 | 0.024 | 0.203 | 0.043 | 0.228 | 0.034 | 0.232 | 0.039 | **<0.0005** | **16%** | **<0.0005** | **6%** | **<0.0005** | **5%** | **0.002** | **-12%** | 0.01 | -14% | 0.379 | -2% |
| **CA1** | 0.045 | 0.005 | 0.035 | 0.008 | 0.041 | 0.006 | 0.042 | 0.006 | **<0.0005** | **24%** | **<0.0005** | **9%** | **<0.0005** | **8%** | **<0.0005** | **-19%** | **<0.0005** | **-21%** | 0.699 | -2% |
| **CA2‎/CA3** | 0.016 | 0.002 | 0.012 | 0.003 | 0.016 | 0.003 | 0.015 | 0.002 | **<0.0005** | **24%** | 0.023 | 4% | 0.012 | 8% | **<0.0005** | **-27%** | **0.001** | **-21%** | 0.154 | 5% |
| **CA4** | 0.018 | 0.002 | 0.013 | 0.002 | 0.017 | 0.003 | 0.016 | 0.002 | **<0.0005** | **27%** | **<0.0005** | **4%** | **<0.0005** | **12%** | **<0.0005** | **-31%** | **<0.0005** | **-21%** | 0.012 | 8% |
| **Dentate gyrus** | 0.021 | 0.003 | 0.016 | 0.003 | 0.021 | 0.003 | 0.019 | 0.003 | **<0.0005** | **22%** | **<0.0005** | **2%** | **<0.0005** | **9%** | **<0.0005** | **-26%** | **0.001** | **-17%** | 0.061 | 7% |
| **Subiculum** | 0.028 | 0.003 | 0.023 | 0.005 | 0.026 | 0.004 | 0.027 | 0.005 | **<0.0005** | **17%** | **<0.0005** | **9%** | **0.002** | **5%** | 0.01 | -9% | 0.021 | -14% | 0.34 | -4% |
| **Presubiculum** | 0.022 | 0.003 | 0.020 | 0.005 | 0.020 | 0.005 | 0.022 | 0.006 | **<0.0005** | **7%** | **<0.0005** | **9%** | **0.003** | **0%** | 0.309 | 2% | 0.174 | -7% | 0.11 | -10% |
| **Hippocampal tail** | 0.040 | 0.005 | 0.039 | 0.008 | 0.040 | 0.009 | 0.043 | 0.010 | **<0.0005** | **4%** | **<0.0005** | **1%** | **0.003** | **-6%** | 0.073 | -2% | 0.076 | -10% | 0.439 | -8% |

**Supplementary Table 2.** Comparisons of volumetry of the hippocampal subfields in 97 healthy controls for scanners with different magnetic field strengths (1.5T versus 3T). p-values denote significance on ANCOVA test (adjusting for age). Bold represents a significant difference between groups after correcting for multiple comparisons.

|  | **1.5T (n=35)** | | **3T (n=62)** | | **1.5 versus 3T** | |
| --- | --- | --- | --- | --- | --- | --- |
| **Structure** | **Mean** | **SD** | **Mean** | **SD** | **p-value** | **%** |
| **Whole hippocampus** | 0.467 | 0.049 | 0.488 | 0.045 | **<0.0005** | **-5** |
| **CA1** | 0.086 | 0.010 | 0.091 | 0.009 | **0.007** | **-5** |
| **CA2‎/CA3** | 0.032 | 0.004 | 0.032 | 0.004 | 0.271 | -1 |
| **CA4** | 0.035 | 0.004 | 0.036 | 0.003 | **0.005** | **-4** |
| **Dentate gyrus** | 0.040 | 0.005 | 0.042 | 0.004 | **0.004** | **-4** |
| **Subiculum** | 0.055 | 0.007 | 0.057 | 0.005 | **0.004** | **-3** |
| **Presubiculum** | 0.042 | 0.006 | 0.045 | 0.006 | **<0.0005** | **-9** |
| **Hippocampal tail** | 0.078 | 0.009 | 0.081 | 0.010 | **<0.0005** | **-4** |
